# Ottorrhœ—Its Rational Treatment

**Published:** 1886-05

**Authors:** J. D. Arnold

**Affiliations:** San Francisco


### OTORRHŒA–ITS RATIONAL TREATMENT.

J. D. ARNOLD, A. M., M. D., SAN FRANCISCO,

OTORRHŒA may be considered as the most *important* and most *neglected* of aural diseases; whether it occur as a disease *per se* or as a symptom of the many affections that endanger the sense of hearing. Bonnafont, after careful and thorough investigations conducted in the deaf-mute asylum of France, declares that fully one-fifth of all deaf-mutes in that country are in this deplorable condition because of otorrhœa. neglected during childhood. This is a terrible indictment against the general practitioner, who, for the most part, regards a "running at the ear" as too trivial a complaint to take under serious advisement. It is well known that among the laity there exists a fixed belief that great harm may result from any attempt to stop a purulent discharge from the ear. As a general thing it is useless to seek the origin of popular superstitions, but the basis of this special one is easy to find; for it happens every day that medical men speak of an otorrhœa as a *salutary suppuration*, and seem to regard it as a *noli me tangere*. Entertaining such views, it is not surprising that a physician, when consulted about a case of this nature, will probably say, "Oh, that is a simple affair, and will get well of itself"; or, if he be pressed for treatment, he may so far relent as to advise an occasional washing out of the ear with warm water. It never enters his head that otorrhœa is always a symptom of some very palpable disease of the auditory apparatus, which should be treated on general surgical principles. It never occurs to him to make a searching examination of the meatus, the tympanum, and the eustachian tube, without which it is impossible to diagnose the case accurately or to treat it rationally. Of course, what I have said is not true of all general practitioners, but certainly is true of the great majority of them. It happens daily to aurists that they are consulted by the parents of children suffering from otorrhœa about the *advisability* of instituting treatment. And when he endeavors to impress upon his hearer the great importance of a cure, he is told the family physician thinks differently, and has said that no harm can come of it, or, perhaps, that "it is better for the matter to come out than remain in the system." Thereupon the specialist is compelled, with such ingenuity as he may possess, to lick the conflicting opinions into some sort of harmony; whilst *mentally* he concludes that either the family doctor does not have the skill to make the necessary examination, or that he is ignorant of the danger of neglecting such cases.

Let me insist again upon the fact, that far from being a trifling ailment, whose treatment may safely be left in the hands of parents or nurses, every *otorrhœa* is a grave affection and demands prompt and discriminating treatment. For, not to speak of the possible implication of the mastoid cells, or the spread of the morbid process to the cephalic membranes, which is nearly always fatal to the patient, the partial or complete destruction of the tympanic membrane, or of the ossicles, is always attended by more or less impairment of hearing.

The causes and complications of otorrhœa are so various that it is impossible in a brief paper like the present to bring them all in review. I shall, therefore, refer only to that class of cases in which the morbid process has its seat in the middle ear. I do this with the less regret, because when the disease is confined to the external ear whether it be due to the presence of a foreign body, the impaction of cerumen, or to a furnuculosis or eczema of the canal, the diagnosis is usually supplied by the patient himself, or becomes evident upon simple inspection, and the indications for treatment are sufficiently obvious. In the greater number of cases the discharge dates from an attack of *otitis media*, an affection so common among infants during the period of dentition, and a frequent sequela of the eruptive fevers of childhood. In more advanced life the otitis is often due to an extension of a catarrh from the pharynx or posterior wall, or to the incautious use of the nasal douche. From whatever cause it arises, the otorrhœa is nearly always chronic when the patient applies for treatment, and often he is uncertain whether the discharge has lasted six months or six years.

The term otorrhœa is, in a certain sense, objectionable; it is at best merely descriptive of a symptom common to many diseased conditions of the auditory apparatus, and should not be made an easy limbo of diagnosis, for it always means more than a *ruuning at the ear.*

In the treatment of simple otorrhœa, the metallic salts so long used, to the exclusion of almost everything else, have fallen into deserved disrepute, and are now only employed in exceptional cases. In all *otorrhœas* uncomplicated by granulations in the tympanic cavity, and where the perforation in the drum is large enough to admit its introduction into the middle ear, the insufflation of finely powdered boracic acid is all that is needed for topical application. Of course a frequent removal of all secretion by the combined use of the aural syringe and the *ustachian catheter* or politzer bag must always precede any local medication of the ear; for in the treatment of this cavity cleanliness above all things is essential to success. When the opening is too small to admit the powder in bulk, a saturated solution of boracic or salicylic acid may be used with advantage. It is my constant practice in these cases, after thoroughly cleaning the tympanic cavity with the air douche, to introduce a minute pledget of borated absorbent cotton close up to the perforation ; this acts as a sort of drainage tube and prevents the stagnation of pus behind the drum. Any case of *otorrhœa*, if uncomplicated by granulations upon the mucous membrane of the middle ear, or disease of the antrum, or by stricture of the eustachian tube, will readily yield to the treatment just indicated. And even when the presence of some vegetable parasite in the ear is at the root of the trouble, the boracic acid treatment suffices for a quick and radical cure, two or three applications being usually enough to destroy completely the mycelium and spores of the common *aspergillus*, whose favorite seat is the external surface of the drum membrane. Sexton and Blake have of late advocated the dry treatment of otorrhœa, using the cotton carrier to remove all secretion instead of the douche. In my experience this plan has proven superior only when there is present an eczema of the meatus, which renders the use of fluids objectionable.

One is, however, sometimes baffled by the persistence of an apparently simple otorhœa, until a more careful examination detects granulations upon the membrane or in the middle ear. The most common seat of such formations is the promontory on the posterior wall of the tympanic cavity. It is not unusual that such formations project through a rupture in the drum, and in such case it is very difficult to determine, even with the sound, whether they spring from the membrane itself or the cavity beyond. The growth is of a vivid red color, and is covered with small granular elevations like a mulberry. If the granulations are of sufficient bulk to fill up the perforation and dam up the pus in the cavity behind it, there is obvious danger of extension of the inflammatory process to the meninges of the brain, which are separated from this part of the ear by only a thin lamella of bone. Indeed, from the anatomy of the part it is surprising such an accident does not happen oftener than is really the case, for the roof of the tympanum is formed by an extremely thin plate of bone, which is often defective in parts and sometimes altogether absent. Toynbee describes several pathological specimens in his possession, in which the head of the malleus projected through an orifice in the vault of the tympanic chamber, and was directly covered by the dura matter. It is clear that under such conditions a chronic inflammation of the mucous investment of the middle ear gains vastly in importance and gravity from the imminent danger of brain complication. Ordinarily, when pus is formed in this region, it finds its way outward through a rupture of the drum, which soon ensues; but the morbid process, if unchecked, favors the formation of warty excrescences upon the mucous membrane, which develop with amazing quickness, fill up the conservative opening in the tympanic membrane, confine the secretions and initiate carious abrasion of the roof of the chamber with all the grave sequelae of such an accident. Nor is the danger of such abrasion confined to this portion of the cavity, for, on its floor, covered only by thing mucous membrane and a shell of bony tissue, which, like the vault, is often defective in parts, lies the internal jugular vein; in the posterior wall lies the *aquœductus* fallopii (sometimes only a groove in the bone), which conveys branches of the facial nerve; on its outer lateral wall is the opening into the antrum and mastoid cells with their intimate relation to the lateral sinus. Remembering, farther, that close behind this cavity the vagus and glosso-pharyngeus make their exit from the skull through the jugular foramen, it becomes apparent that danger to many important structures lie thick around this minute bit of anatomy—the middle ear. I once heard Brucke say, "Any pathological process in this seemingly insignificant space is like a breath of wind toying with the lid of Pandora's box." When the rupture of the drum has not yet taken place, and the secretions are thin and serous, they readily find vent into the pharynx through the eustachian tube, and thus the danger of accumulation and decomposition is avoided. But often this small conduit becomes impervious, for in many cases it is implicated before the tympanum itself.

Besides the fact that a persistent discharge from the auditory canal should always arouse suspicion, there are few symptoms that indicate the presence of granulation in the middle ear. Pain is rarely felt after the membrane is once ruptured, unless the mastoid cells are already implicated.

Direct inspection of the parts is absolutely essential to the diagnosis and treatment of ear disease, and the management of the reflector and speculum is so easily acquired that every physician should be able to use them. at least for diagnostic purposes.

For the removal of polypoid growths in the external meatus the wire snare is most frequently used, and practically leaver little to be desired. The galvanic-cautery loop has several unquestionable advantages over the cold wire. It renders the operation almost painless and at the moment of removing the growth destroys its base. But these instruments are by no means suitable for granulation upon the drum or within the tympanic cavity: here the ring-knife or curette is the most useful and sufficient instrument. The objection to the use of *silver nitrate* for caustic purposes in the middle ear is its tendency to form albumenoid flocculi, which are removed with difficulty and act as foreign bodies. When the growths are so situated as to be inaccessible to an instrument, or when the patient will allow no operative procedure, the local use of absolute alcohol is of excellent service. This should be instilled warm into the ear and allowed to remain at least five minutes. The application should be made once or twice each day. As I have stated above, pain is seldom a symptom of these cases; when it is present it can be allayed by the frequent use of a one per cent. sol. sulphate atropia, or, better still, a five per cent. sol. muriate cocaine.—*South California Practition.*

